# Childhood disability in Turkana, Kenya: Understanding how carers cope in a complex humanitarian setting

**DOI:** 10.4102/ajod.v5i1.277

**Published:** 2016-09-29

**Authors:** Maria Zuurmond, Velma Nyapera, Victoria Mwenda, James Kisia, Hilary Rono, Jennifer Palmer

**Affiliations:** 1 International Centre for Evidence in Disability, London School of Hygiene & Tropical Medicine (LSHTM), London, UK; 2 Kenya Red Cross Society, Nairobi, Kenya; 3 London School of Hygiene and Tropical Medicine, and Opthalmologist, Kitale, Kenya; 4 Department of Infectious Diseases Epidemiology, London School of Hygiene & Tropical Medicine, UK; 5 Centre of African Studies, School of Political & Social Sciences, University of Edinburgh, London, UK

## Abstract

**Background:**

Although the consequences of disability are magnified in humanitarian contexts, research into the difficulties of caring for children with a disability in such settings has received limited attention.

**Methods:**

Based on in-depth interviews with 31 families, key informants and focus group discussions in Turkana, Kenya, this article explores the lives of families caring for children with a range of impairments (hearing, vision, physical and intellectual) in a complex humanitarian context characterised by drought, flooding, armed conflict, poverty and historical marginalisation.

**Results:**

The challenging environmental and social conditions of Turkana magnified not only the impact of impairment on children, but also the burden of caregiving. The remoteness of Turkana, along with the paucity and fragmentation of health, rehabilitation and social services, posed major challenges and created opportunity costs for families. Disability-related stigma isolated mothers of children with disabilities, especially, increasing their burden of care and further limiting their access to services and humanitarian programmes. In a context where social systems are already stressed, the combination of these factors compounded the vulnerabilities faced by children with disabilities and their families.

**Conclusion:**

The needs of children with disabilities and their carers in Turkana are not being met by either community social support systems or humanitarian aid programmes. There is an urgent need to mainstream disability into Turkana services and programmes.

## Background

### Disability in humanitarian crises

The World Report on Disability estimates that 15% of the world’s population experience some form of disability, and an estimated 93 million children aged 0–14 years are living with a moderate or severe disability. The majority are in low- and middle-income countries (LMICs; World Health Organization 2011). The same report highlights that conflict and natural disasters can both cause disabilities and make people with existing disabilities even more vulnerable. Humanitarian crises are social and material environments which may exacerbate the vulnerability of people with disabilities because of a breakdown in social services (medical and education), a reduction in income support for food and other basic necessities, diminished mobility and opportunities for migration and the loss of carers (Kett & van Ommeren 2009; Reilly 2010). Article 11 of the Convention on the Rights of Persons with Disabilities emphasises measures to protect the safety of people with disabilities during armed conflict and natural disasters (UN 2006). Additionally, a number of guidelines exist to support the mainstreaming of disability in humanitarian interventions (Handicap International 2005; Sphere 2011). Within a low-resource context, community-based rehabilitation (CBR) is also an important recommended strategy for rehabilitation, poverty reduction and the social inclusion of people with disabilities that is relevant to humanitarian contexts. This approach recognises the central role of the family and their communities, as well as relevant government and non-governmental organisations, in reducing disability (World Health Organization 2010).

Humanitarian crises such as those caused by armed conflict and natural disaster inevitably create new social processes, not all of which lead to harm. Crises may reinforce a community’s sense of identity and systems of coping (Hodgson 2000) or prompt exposure to global humanitarianism and associated liberal systems and concepts (Grabska 2014). Indeed, people with disabilities in some highly managed settings (such as refugee camps) experience access to humanitarian programming as liberating, not only because such programmes meet everyday accessibility needs such as by providing latrines that are easy to use, but also by helping transform displaced populations’ attitudes towards disability (Mirza 2013). As in non-crisis affected contexts, disabled children and their families in humanitarian and post-humanitarian settings may face multiple barriers to social inclusion. These barriers include increased caregiving duties, which prevent participation in economic activities, and poor informational access to or discriminatory exclusion from services that mitigate vulnerabilities (Miles & Medi 1994; Ngo *et al*. 2012). The needs for informal care and CBR in crisis and post-crisis settings also likely impact women more than men (Berghs 2015). There is, however, a need for better understanding of the lived experiences of adults and children with disabilities during humanitarian crises; particularly, little is known about family coping strategies in such settings.

### Coping in a complex humanitarian context in Turkana

Turkana County, in north-west Kenya, has been classified at various points over the last several decades as suffering from a complex humanitarian crisis because of multiple causes of vulnerability which compound each other. The climate is arid and suited for livestock production, which supports 60% of the population (Boulton 2012), but is subject to periodic drought. Rains are not only erratic with frequent total failure, but they also produce flash flooding. Within Kenya, Turkana is the largest county geographically and one of the most historically under-developed; people who live there suffer from some of the poorest schools, roads and health services in the country, a situation which has been unable to reverse by long-standing (but often poorly funded) government and humanitarian interventions (Broch-Due & Sanders 1999). Health and rehabilitation services are limited to the county hospital and a small, fragmented network of faith-based clinics and hospitals.

Poverty levels are 20% above the national average with per capita livestock wealth declining and dependence on food aid increasing (Kenya National Bureau of Statistics 2014). The large majority (78–94%) of households in Turkana experience problems accessing food (Ochola 2011). In recent years, up to 15% of the population of Turkana has been served by United Nations ‘food assistance for assets’ programmes (food in return for work on projects that increase a community’s resiliency), and the general acute malnutrition rate among children has hovered at around 15%, the threshold between a ‘serious’ and ‘critical’ nutritional emergency (Office for the Coordination of Humanitarian Affairs 2013). With the proliferation of small arms seen over the last several decades, livestock raiding also contributes to famine, inter-communal violence and displacement and harms social systems for coping (Hendrickson, Armon & Mearns 1998). Consequently, in Turkana, as in other pastoralist areas of Africa, low-level, chronic violence and the politics of marginalisation reproduce one another (Pike *et al*. 2010).

In such a setting, pastoral mobility, including crisis-induced displacement, largely configures Turkana people’s access to food, social services and income (Pike *et al*. 2010). Turkana social institutions (such as the maintenance of land rights and systems of livestock redistribution during crisis) remain key for people to manage the environmental precarity of living here (McCabe 1990), but they tend to exclude families living in towns, making them more dependent on cash economies or aid (Broch-Due & Sanders 1999). As carers, women play a key role in upholding these social institutions which contribute to community resilience. A good example of the instrumentality of pastoralist women in northern Kenya is in the area of nutrition (Pike *et al*. 2010). Access to milk from livestock is a particularly important determinant of nutrition and therefore health. When crisis forces men to move with cattle, this puts women and children at a nutritional disadvantage. To counter such vulnerability, pastoralist women therefore practise nutritional buffering of children’s diet in an age-related pattern of communal moral responsibility; older women buffer younger mothers and all women forego food when children are hungry. Women in the Turkana are also more likely to be in a polygamous union (21%) compared with the national average of 10.2% Kenyan average (Kenya National Bureau of statistics 2010), and crisis can put a particular strain on the resources of polygamous families, with family breakdown (Wawire 2003).

The results of a recent study of childhood disability and malnutrition in Turkana are therefore troubling, which found that children with disabilities are more likely to be malnourished than their neighbours and even their siblings (Kuper *et al*. 2015). This vulnerability could reflect difficulties that carers of children with disabilities have in accessing traditional social support systems and/or humanitarian interventions. As has been shown in post-conflict Mozambique (Miles & Medi 1994), for families already stressed from the effects of war, poverty and drought, having a disabled child can exacerbate an already desperate situation, with the family invariably becoming poorer.

In this study, we present a picture of the lived experiences of carers of children with disabilities living in an ongoing, complex humanitarian crisis in Turkana, Kenya. We use the World Health Organization’s International Classification of Functioning, Disability and Health (ICF), a biopsychosocial model of disability that synthesises a medical and social model of disability. This framework outlines the dynamic relationship between the impairment or health condition with activity limitations and participation restrictions, and the mediating role that environmental and personal factors can play (World Health Organization 2001). We pay particular attention to carers’ daily needs, priorities and coping strategies as well as the social processes which permit or limit opportunities for them to access services and participate in community life.

## Methods

### Study setting and sample

This research was conducted in September 2012 and May 2013 in Lodwar, the county’s largest town, and surrounding areas of Turkana Central District (now a sub-county) in collaboration with the Kenya Red Cross Society (KRCS). An initial scoping visit was undertaken to map disability-related services and aid programmes in Turkana county (September 2012). Turkana Central District was selected for this study and for a later survey of childhood disability prevalence for several criteria: it supports a range of livelihood types (pastoralism, fishing, agriculture, small businesses), was relatively safe and KRCS was present in the district to facilitate access to families caring for children with disabilities. The survey, conducted Jul–Aug 2013, estimated a minimum prevalence of moderate-to-severe disability in children at 0.75% (0.66–0.83%), with the true prevalence likely to be much higher (Kuper *et al*. 2015). Physical impairment such as cerebral palsy, rickets and muscular dystrophy was the most prevalent form of disability followed by epilepsy, visual, hearing and intellectual impairment; congenital causes of the disability were most common. The majority of children (85%) had never received any form of rehabilitative service such as therapy/exercises, assistive devices, surgery or occupational advice.

For our qualitative interviews, a list of children with disabilities living in the Turkana district aged 10 years and younger was collated from existing KRCS disability project data; additional information on demographics and the type of impairment was provided by KRCS CBR workers and local community health workers. Children were purposively sampled to cover a range of ages (1–5 years and 6–10 years), genders and impairments (physical, visual, hearing and intellectual). As the research was conducted during the rainy season some adjustment had to be made to the final choice of villages to take into account accessibility for the survey team and resulted in the exclusion of the most isolated and inaccessible villages. Cattle-rustling and tribal conflicts also prevented access to some districts at this time.

Carers were interviewed from 31 households, providing information on a total of 36 children with disabilities, of which 25 were boys and 11 girls. Eight children were in school. Of the carers interviewed, 19 were mothers, 3 grandmothers, 3 grandmothers and mothers (combined), 2 siblings, 4 fathers and 1 mother and father (combined). Only the men did not self-identify as the primary carer. A quarter of the families interviewed had recently migrated: two were pastoralists who regularly moved with livestock, five were displaced by flooding, drought or cattle raiding. Families in the sample were drawn from 23 villages, in three divisions, four locations and seven sub-locations.

To contextualise information collected from carer interviews, interviews were conducted with 16 key informants including village elders, hospital- and clinic-based staff, community health workers and teachers. Furthermore, two focus groups were conducted in two sites (where unplanned groups of carers had congregated when they heard about the research), and individuals were selected from these groups for one-to-one interviews (Table 1).

**TABLE 1:** Sample of children (*n* = 36).

Child disability	Male	Female	Total
Physical	8	5	13
Sensory (hearing or visual)	2	2	4
Intellectual	2	0	2
Multiple	13	4	17

### Data collection and analysis

Interviews were semi-structured and conducted by two researchers (M.Z. and V.N.) through translation between Turkana or Kiswahili and English. At the end of each day the project team met to review all interviews, to discuss and agree on key emerging issues and to identify any gaps that necessitated further exploration and additional questions in follow-up interviews. Detailed notes were taken during all interviews, and all interviews were also recorded and transcribed into English.

Most interviews with carers took place at the child’s home, unless roads were impassable, in which case interviews were undertaken at a nearby health centre. Given that most family dwellings were very small, it was often impossible to have privacy for the one-to-one interviews, and some interviews were inevitably conducted with other family members present. Interviews covered the following areas: beliefs and attitudes about the child’s condition, the impact of caring for a child with a disability, nutrition and feeding practices and factors which impacted upon children’s access to services (health, education and humanitarian programmes including nutrition programmes). Interviews with key informants and focus group discussions focused on the availability of services, referral procedures and barriers to inclusion of children with disabilities in social systems.

Both interviewers separately identified a list of key themes and sub-themes through an iterative process, and these were then discussed, refined and cross-checked for consistency to provide an overall thematic coding framework for systematic analysis by M.Z. after fieldwork, by using NVivo 10 software (Green & Thorogood 2009).

### Ethical consideration

This project received ethical approval from Moi Teaching Hospital in Kenya, and from the London School of Hygiene and Tropical Medicine in the UK. Families were contacted and visited by local KRCS volunteers in advance of the interviews to provide clear verbal and written information about the research, and signed consent was obtained for all interviews. One component of the ethics protocol was for referrals to be supported by the KRCS team following the interviews. Quotes from respondents have been anonymised to protect their privacy.

## Findings

### The gendered impact of caregiving and livelihoods

In a context of widespread poverty, scarcity of services and poor infrastructure, it was evident from all interviews that having a child with a disability impacted the whole family, generating specific challenges related to caring, income generation and the psychosocial health of carers. Impacts were most notable on women, who are the primary carers in families in Turkana. Mothers, grandmothers and female siblings typically cared for the children in our sample. In the larger survey, children with disabilities were significantly more likely than neighbour controls to have a female head of household, and half of the households we interviewed were single parent, female-headed. Reasons given for a father’s absence, or for spending a long period away from the home, commonly involved stigma related to the child’s disability, with very little, if any, family support provided by the father following the birth of a child with a disability. These reactions compounded the gendered burden of caregiving and the poverty of the family.

Caregiving arrangements for ‘P’ are illustrative of the complexity of these issues. P is a 10-year-old boy who was intellectually impaired and had epilepsy; he lived with his mother and six siblings, and his oldest sister had been taken out of school to help care for P. His illness began when he was about 6 years old; his family sought treatment for him at the local health centre and spent a considerable amount of money on seeking a cure from a traditional healer. His father subsequently left the family and stopped providing any support. The family reported they were not in receipt of any community or humanitarian support and the psychosocial impact of this on P’s mother was clear:

‘I think someone bewitched the father of the chid, and then the spell went to the child.… They told me [at the health centre] to take the child home and seek the help of the traditional healer … my husband and I spent a lot of money.… When my son’s condition worsened I felt desperate, I lost hope, and I know there is nothing I can do to heal my son.’

The impact of increased caregiving responsibilities on being able to pursue livelihood activities was a key issue for families, and particularly in, but not exclusive to, female-headed households. A mother from another single female-headed household described the frustration she felt of balancing her caregiving roles with income-generating activities:

‘I am not able to do any work here all day. I have to carry the child all through the day. I am not able to make as many mats as other women make. I only make one mat every month while other women make even 5 mats. Sometimes I am not able to make any mats at all.’ (Mother of 6-year-old boy with cerebral palsy)

The need to work often resulted in a child with a disability being left on their own for several hours during the day. One grandmother in a peri-urban context described how her grandson was often left on his own under a tree. The child had severe cerebral palsy and was immobile, without even an assistive device to sit on, and without support from close neighbours: ‘When all the children have gone to school he is left alone. The mother might be in town and I might be collecting firewood or on the farm’.

For displaced families, the absence of support from extended family also accentuated the challenge of combining caregiving and livelihoods. One mother of an 8-year-old boy who had Down’s Syndrome and was visually impaired described moving to a new village after her husband had been killed in a livestock raid. Although neighbours offered occasional support, working was difficult with no family nearby to help:

‘He is with me throughout the day. It is so difficult … when I have to work I sometimes lock him in the compound, and sometimes I take him with me [*to collect firewood*]. When he is locked inside alone he runs around and he cries. Sometimes neighbours will come and comfort him and he will sleep.’ (Mother of an 8-year-old boy with Down’s Syndrome)

### Beliefs about the causes of disability

Beliefs and attitudes linking disability and misfortune influenced parents’ decisions to engage with services and seek treatment, as well as the type of treatment sought. Carers had very low levels of biomedical understanding about their child’s impairment, and treatment was most commonly sought from traditional healers. In the small number of instances where families had visited a hospital or clinic, there was still considerable confusion about their child’s condition, the cause and options for treatment.

While a very small group of parents provided a biomedical rationale for the disability, some carers suspected witchcraft as in the case of P above; ‘God’s will’ and the intervention of ancestors were also common justifications. Moreover, many families held pluralistic views on the causes of the disability, whereby many possible reasons were considered simultaneously. Non-biomedical explanations cast disability as a misfortune typically interpreted in the context of problematic interpersonal relationships germane to the sociopolitics of the Turkana region, as in the following explanation:

‘Some say that it is God who is annoyed with them. Others say that their forefathers were disabled, and it is a replica of that [*Interviewer: Can you explain more?*] In the past people went for raid and in the process of raiding they might have killed a disabled person, and so God now makes sure that they have got that disability.’ (Mother of a 7-year-old girl with a hearing impairment)

As shown by others, intervention by ancestors, God and witches/wizards is a common rationale for childhood illness and disability in Turkana (Shelley 1985).

In communities close to Lake Turkana, poor natural environmental conditions such as salty water or soil were also commonly believed capable of causing disability. Yet, despite some carers’ associations between health problems and the lake, the lake remained an essential source of livelihood. Disability therefore appeared to be interpreted as an unfortunate trade-off and inevitability of living near the lake, and commonly no diagnosis or treatment was sought. As one single parent mother explained, in relation to her daughter’s condition:

‘People say the salty water from the lake and the sandy soil makes the bones weak, especially because it is difficult to walk in sand. Some people in the community call it ‘agule’ [*polio*], others call it ‘lotoro’ - a condition caused by the water in the environment and soil … I have been considering taking her to the hospital but I have no faith that anything can be done medically about this disease; it is caused by the environment.’ (Mother of a 5-year-old child with a physical impairment)

### Stigma and the absence of support

Stigma and shame associated with having a child with a disability was pervasive and influenced how children were cared for, the wider support available from the community, and acted as a key barrier to accessing services and aid.

One mother, whose son of 8 years was completely blind and spent most of his day inside the house, illustrated this profoundly, saying ‘I do not think ‘S’ is alive. I am just taking care of him until his final death’.

Children with disabilities were seen as a burden because they could not help with household chores or work with the livestock, all of which were important roles in this rural and pastoralist setting:

‘I do not think that this child is of benefit to the family in any way. She just remains to be counted as part of my household. She can’t get married or help with household chores.’ (Mother of a 6-year- old girl with a physical and intellectual impairment)

Stigma also prevented children from accessing services. One grandmother explained how she had never taken her 10-year old grandchild with multiple disabilities out of the compound to seek help or to register him for any type of aid project: ‘My child has not been part of any food programme, but we haven’t exposed him to any of these programmes. [*Interviewer: Why?*] I was ashamed to take him out’.

While some parents commented that their family or community did not overtly stigmatise their child, and some benefited from material support, they nevertheless felt shame in the absence of family or community social support. For instance, one single mother highlighted her pain from the fact that her sister refused to carry her child:

‘I feel bad having to carry the child around by myself and no one is there to help me. My sister does not want to help me carry my child. She provides for us and helps me a lot with money and food. But she doesn’t want to associate with my child because he is disabled. She does not carry him at all. That is not all; his father refused to take responsibility over his child because the child is disabled.’ (Mother, with son of 3 years with cerebral palsy)

Key informants talked about the absence of children with disabilities in their services because the children are hidden. One village chief, discussing this point, argued that while disabled children in his village were not necessarily hidden, they were largely kept at home in the absence of clear support or knowledge of what treatment or rehabilitation might offer:

‘I was surprised when Kenya Red Cross Society brought all the disabled together in one point. I was surprised how many I saw. They are not being hidden, but they are kept at home unless they know that something can be done with their situation.’

### Caregiving in an arid rural environment

The limited mobility of children was among the greatest challenges for carers in the arid Turkana environment where carers must carry disabled children while covering long distances to collect firewood and water. The absence of assistive devices as well as the difficult terrain for a wheelchair are additional challenges. Toileting and the personal care of children, particularly when they were incontinent, was also a major problem given the absence of latrines and huge difficulties around access to water. This challenge was highlighted by key informants and carers:

‘I am confined to caring for him all day. … You know, once somebody is lame or disabled and the family has no way to solve the problem, it becomes a desperate situation … I am the one who cleans him up when he passes stool. I also take him out to pass his stool. This is very stressful but I have no way out.’ (Mother of son of 10 years with epilepsy and intellectual impairment)

In one village, this problem was exacerbated when the communal pump was damaged following flash flooding, and the mother described needing to resort to a 4-h round trip to dig a shallow well at the nearest river bed, which is of course a challenge for all families, but it was magnified for her when carrying an older, heavy child with a disability. While some carers elected to leave children behind during water collection, others worried about the consequences of doing so, as one mother of a daughter with multiple disabilities explained:

‘For me being with her most of the day is very difficult for me to bear. But I fear that if I leave her alone she may fall in the fire and this thought traumatises me, so I am forced to stay and care for her all day.’

Long distances to access healthcare was also a particular challenge for a mother recently displaced by flooding to a remote area. Although her 8-year-old daughter with epilepsy and an intellectual impairment was ill almost every month, it was rare that she could leave the rest of her duties to carry her daughter the 4 km to the nearest dispensary.

The difficult environmental conditions for all families in Turkana were furthermore raised by carers as a reason why other families may be reluctant to share the burden of caring. As described by this father of a boy with hydrocephalus and polio, ‘Some community members support the children whenever they can. … The majority, however, do not support them at all. In the community everyone fends for himself’.

### The challenges of accessing services

There were few projects in Turkana designed to serve people with disabilities (Office for the Coordination of Humanitarian Affairs 2013) and limited government or faith-based rehabilitation services even within the main county hospital, making it difficult for families to access assistive devices. The limited dedicated services is within a wider context of limited health service provision in Turkana, for example 19 nurses per 100 000 population compared to 55 nationally (Government of Kenya 2014). With the exception of eye health services, key informants highlighted that referral processes for treatment or rehabilitation of impairments were complex or absent in Turkana. There was a lack of information about the limited rehabilitation services which were available, and a wider lack of knowledge about disability amongst community-level healthcare professionals (Merlin 2012).

The large distances, the remoteness of villages and very limited transport meant there were very substantial opportunity costs, in the form of time lost away from work, for families who chose to seek out rehabilitation services.

‘We had to stay in Lokichar for a month for the child to be assessed and given treatment … we are business people; our long stay in Lokichar made our business not flourish. We also lost time and money in seeking treatment.’ (Father of a girl with a physical disability)

Almost every carer identified costs as a barrier to accessing healthcare, and sometimes important assets such as livestock were sold off to pay for ongoing treatment or the constant quest for a ‘cure’ for the disability, which included use of traditional healers. The impact on families of paying for services could be substantial because in many cases the carers commonly described their child with a disability as being more frequently ill, compared with siblings:

‘We are unable to save any money for the future because almost everything is spent on the treatment of our child.… You know when the child is well one can afford to save money for other things and for the children’s education.’ (Father of 3-year-old boy with a physical impairment)

In some families, the lack of income precluded seeking any health services, as explained by one single mother whose son was frequently ill: ‘If he is ill, I boil water and bathe him in water, but I have no money to take him to the health centre’.

In terms of humanitarian aid programmes, there was also confusion and perceptions of unfairness around accessing nutrition programmes among parents - a key need given that most families reported difficulties in providing one meal a day for all family members. School feeding programmes are a common nutrition intervention in Turkana, for example, but most children with disabilities interviewed were not in school and were thus not able to benefit in the same way as their siblings. Others were too young for school and yet were also not accessing food supplementation programmes, as one mother explained:

‘I have not received any help so far. We are still waiting for aid. My sister’s children however … [*receive*] maize and beans in school.’ (Mother of a 3-year-old boy with cerebral palsy)

Likewise, parents faced major practical challenges accessing ‘food assistance for assets’ programmes and food distributions, as the following quotes illustrate:

‘There is a food-for-work programme within the area, but I’m not a beneficiary. There is no way I can leave the child and go to work.’ (Mother of girl of 8 years with cerebral palsy)

‘I used to carry my child across the lake … where distribution of food used to take place. I would pay for a bicycle to transport the food to the lake shore, then put it on a boat and cross over. It is much easier for parents without children with special needs. For instance, my sister used to carry the food by herself, she didn’t need help [*requiring her to pay for additional transport*].’ (Mother of boy of 5 years with a physical impairment)

## Discussion

This research sought to explore the lived experiences of families who care for children with disabilities in the complex humanitarian environment of Turkana, with the overall aim of improving their inclusion in programming and policy. As described by the World Health Organization’s model of disability (World Health Organization 2001), this study highlights how the multifaceted humanitarian context in Turkana magnifies the disabling impact of children’s impairment on them, and also on the carer burden, through a variety of environmental, social and cultural factors which compound the vulnerability of the family. Our study also confirms many of the challenges faced by people with disabilities cited in the small but growing body of literature about disability in humanitarian contexts, which include the disruption of social support networks and dearth of supportive services (Lange 2015; Oosterhoff & Kett 2014; Scherrer 2015; Tomlinson & Abdi 2003).

Our study paints an often harrowing picture of the daily lives of children with disabilities and their families. Arguably many families in Turkana face extreme poverty, and access to basic healthcare for everyone is a challenge in pastoralist zones (Pike *et al*. 2010; Sheik-Mohamed & Velema 1999). Poor roads, large distances, few services and limited transport affect everyone’s access to services; however, this problem is magnified for children with disabilities, who often need to be physically carried long distances, or complex transport arrangements need to be made. Our findings mirror those of an Australian study which describes the mobility restrictions of disability on top of the difficulties for dispersed populations to access services as a ‘double disadvantage’ (Gething 1997). In the drought-stricken environment of Turkana, water scarcity is a vital issue for everyone (London School of Hygiene and Tropical Medicine 2013), but the challenges are augmented in a household with a child with a disability who may need to be carried for water collection and may have additional self-care needs. Reviews of water and sanitation issues for persons with disabilities highlight the critical importance of these issues, yet they are often overlooked in programmes which are not disability inclusive (Danquah 2014; Groce *et al*. 2011).

It is well recognised that family and community support networks are essential in the care of children with disabilities in many low-resource settings, in particular where there is a paucity of services (World Health Organization 2011), and yet in emergency contexts, family and social networks are often weakened or destroyed (Oosterhoff & Kett 2014). Our findings emphasise the particular isolation of carers in this disability context and the limitations of community support mechanisms. Fathers were absent in more than half of families, for example, and the stigma of having a disabled child was offered as a common explanation. When even women’s sisters would not touch a child with a disability to share the burden of carrying them during chores, this particularly heightened female carers’ sense of social isolation. Although we could not investigate it in-depth, social isolation of women carers could also affect other crisis-related coping mechanisms, such as nutritional buffering. Stigma is a complex phenomenon often linked to the cultural context and associating disability with witchcraft or supernatural intervention is pervasive in many contexts, including in Turkana ( Shelley 1985; Van Brakel 2014; World Health Organization 2011). In the specific region of Lake Turkana a recent environmental study highlighted reportedly high levels of skeletal ‘deformities’ linked to changing salinity levels, and a persistent local view that the deformation was a curse (Avery 2013). This behaviour which normalises a state of ill health and long-term suffering because of structural inequalities can also be common among marginalised groups in the region (Sundal 2009).

However, there are few studies which have also explored the specific impact of stigma on carers and caregiving. In our study, such stigma limited not only carers’ access to support from extended families or communities, but also their access to government, NGO services and humanitarian programmes, which was because local social support is often needed to offset the opportunity costs involved in seeking formal services and programmes, such as providing care for children left behind whilst services are accessed or the costs of transportation when walking while carrying a heavy child is impossible.

This isolation of carers, in turn, impacts on children’s access to healthcare and carers’ access to livelihood opportunities. Several studies have indicated that children with disabilities in LMIC settings are more likely to have problems with serious illnesses or malnutrition (Groce *et al*. 2013; Tompsett, Yousafzai & Filteau 1999; Yousafzai, Filteau, & Wirz 2003). Our qualitative study corroborates the findings of the prevalence survey in Turkana which showed that children with disabilities were more malnourished but at the same time less likely to access feeding programmes (Kuper *et al*. 2015). Families in this setting also appear to face major challenges meeting the healthcare needs of their children with disabilities; limited service availability, a lack of information about rehabilitation service options and complex referral processes exacerbate this. The challenges of increased caregiving responsibilities on livelihoods in the context of HIV has been extensively described in the literature (Opiyo, Yamano, & Jayne 2008), but there is limited comparable evidence within the disability literature, and what little there is typically limited to studies of adults with disabilities. In humanitarian contexts, food-for-work programmes are intended to benefit the whole community and particularly attempt to target the most vulnerable, and yet our study indicates that carers of children with disabilities are often excluded from such programmes.

In conclusion, a multiplicity of factors compound the vulnerability of children with disabilities and their carers in the complex humanitarian context of Turkana. Our interviews illustrate that children and their carers in Turkana are falling through the safety nets of both community social support systems as well as humanitarian aid programmes established to assist the most vulnerable; this emphasises the urgent need for improved mainstreaming of disability, as well as targeted approaches for inclusion, for example, in terms of how these children can be included in nutrition and food assistance programmes. The intersectionality of gender and disability also needs some consideration given the gendered nature of caregiving. For example, livelihoods programmes need to be more gender-sensitive and more actively inclusive of families with children with a disability who may be ‘invisible’ to authorities, health and humanitarian workers in this setting. As called for by others, disability must be seen as a ‘mobile cross-cutting issue’ that can contribute to social injustice and should not be treated as a narrow ‘specialist medical issue’ (Berghs 2015).
